# GhKWL1 Upregulates *GhERF105* but Its Function Is Impaired by Binding with VdISC1, a Pathogenic Effector of *Verticillium dahliae*

**DOI:** 10.3390/ijms22147328

**Published:** 2021-07-08

**Authors:** Yang Chen, Mi Zhang, Lei Wang, Xiaohan Yu, Xianbi Li, Dan Jin, Jianyan Zeng, Hui Ren, Fanlong Wang, Shuiqing Song, Xingying Yan, Juan Zhao, Yan Pei

**Affiliations:** Biotechnology Research Center, Southwest University, No. 2 Tiansheng Road, Beibei, Chongqing 400716, China; mtchy2008@email.swu.edu.cn (Y.C.); selenazm@swu.edu.cn (M.Z.); wl407002131@email.swu.edu.cn (L.W.); yuwen970526@email.swu.edu.cn (X.Y.); lxianbi@swu.edu.cn (X.L.); jindancj@swu.edu.cn (D.J.); zengjianyan@swu.edu.cn (J.Z.); renhui@email.swu.edu.cn (H.R.); wfl2015@email.swu.edu.cn (F.W.); shuiqingsong@126.com (S.S.); yxingying@swu.edu.cn (X.Y.); zhaojuan0920@swu.edu.cn (J.Z.)

**Keywords:** kiwellins, *Verticillium dahliae*, GhERF105, VdISC1, cotton

## Abstract

Verticillium wilt, caused by *Verticillium dahliae*, is a devastating disease for many important crops, including cotton. Kiwellins (KWLs), a group of cysteine-rich proteins synthesized in many plants, have been shown to be involved in response to various phytopathogens. To evaluate genes for their function in resistance to Verticillium wilt, we investigated KWL homologs in cotton. Thirty-five *KWL* genes (*GhKWLs*) were identified from the genome of upland cotton (*Gossypium hirsutum*). Among them, GhKWL1 was shown to be localized in nucleus and cytosol, and its gene expression is induced by the infection of *V. dahliae*. We revealed that GhKWL1 was a positive regulator of *GhERF105*. Silencing of *GhKWL1* resulted in a decrease, whereas overexpression led to an increase in resistance of transgenic plants to Verticillium wilt. Interestingly, through binding to GhKWL1, the pathogenic effector protein VdISC1 produced by *V. dahliae* could impair the defense response mediated by GhKWL1. Therefore, our study suggests there is a GhKWL1-mediated defense response in cotton, which can be hijacked by *V. dahliae* through the interaction of VdISC1 with GhKWL1.

## 1. Introduction

Cotton, the leading fiber crop of the world, is widely planted in more than 80 countries [1]. However, cotton production is limited by biotic stresses, of which the most serious one is Verticillium wilt caused by members of the fungal genus *Verticillium* [2,3,4]. *Verticillium* has a broad host range of more than 300 woody and herbaceous plant species. *Verticillium dahliae* and *V. alboatrum*, among the most notorious species, cause billion-dollar losses annually worldwide. The soil habitat and their long-term survival for years make control of the pathogen difficult, and thus, Verticillium wilt is the focus of extensive research and management attention [2,5].

During infection, *Verticillium* spp. produce a battery of proteins, including wall-degrading enzymes, transcription factors, and membrane receptors, to facilitate host colonization, often by disturbing host immunity [6]. Putatively, *V. dahliae* secretes more than 700 proteins, also known as effectors [7], which either emphasize pathogenic virulence by overcoming plant defense responses or are directly/indirectly recognized by host surveillance systems, leading to effector-triggered immunity [8]. Some of these effectors interfere with host hormone signaling, by which the pathogens can use the host resources to their advantage [9,10]. For example, in the process of colonization, *V. dahliae* produces VdISC1, an unconventionally secreted isochorismate mutase that converts isochorismate into 2,3-dihydro-2,3-dihydroxybenzoate (DDHB), to inhibit the biosynthesis of salicylic acid (SA) by greatly reducing the accumulation of the SA precursor isochorismate [11] and thereby downregulates the plant innate immune response against biotrophic pathogens [12].

Sustained efforts have been made to identify effective approaches to modify plant resistance to Verticillium wilt. For example, in cotton, the overexpression of various proteins and small RNAs, such as thioredoxin GbNRX1, major latex protein GhMLP28, laccase GhLAC1, lysin-motif receptor kinases (GhLYK1 and GhLYK2), microRNAs (miR166 and miR159), and transcription factors (GhbHLH171, GbWRKY1, GhMYB108, and GbERF1), is reported to be able to enhance plant resistance to Verticillium wilt [13,14,15,16,17,18,19,20,21,22]. Among these proteins, ethylene response factors (ERFs), belonging to the superfamily of APETALA2 (AP2)/ERF transcription factors, have been implicated in regulating the plant defense response [15,23,24,25,26,27,28]. Plants with changed expression of *ERF* genes display altered disease susceptibility, which is associated with transcriptional regulation of defense-related genes, such as *PDF1.2* and pathogenesis-related (*PR*) genes [29,30,31,32,33,34]. Increasing evidence has revealed that *ERF* genes are involved in resistance to Verticillium wilt in cotton [15,17,35,36,37]. The expression levels of *PDF1.2* and *PR5* genes are remarkably reduced in *GhERF6*-silenced cotton plants [15]. The overexpression of *GbERF1-like* improves Verticillium wilt resistance in both cotton and Arabidopsis, and the expression of *PR3* and *PR4* genes is upregulated in *GbERF1-like*-overexpressing cotton plants [35].

Kiwellins (KWLs), a group of cysteine-rich proteins that are among the major protein components of kiwifruit (*Actinidia chinensis*) [38,39,40], were initially identified as a novel substance that causes food allergic reactions [38,41,42,43]. The expression of *KWL* genes is highly upregulated during the plant response to pathogenic infection and whitefly infestation [7,44,45,46,47]. Recent research has revealed that two paralogs in maize (*Zea mays*), *ZmKWL1* and *ZmKWL1-b*, function redundantly in smut disease resistance [48,49]. ZmKWL1 disables the effector UmCmu1, which can prevent the biosynthesis of salicylic acid through physical interaction and, in turn, secures the plant defense response [48]. Similarly, ZmKWL1-b, the closest homolog to ZmKWL1, is able to interact with UnCmu1 and considerably reduce the pathogenicity to the plant [49]. However, given their obscure functions and absence in the Brassicaceae [48], including the model plant Arabidopsis, the roles of KWL family members are poorly understood.

In this study, we identified multiple *GhKWL* genes in the upland cotton genome. Among the pathogen-inducible *GhKWLs*, GhKWL1 was localized in the nucleus and positively regulated plant resistance to *V. dahliae* infection through an ERF-mediated pathway. In turn, VdISC1, an effector of *V. dahliae*, could interact with GhKWL1 to attenuate the GhKWL1–GhERFs-mediated plant defense response. Overexpression of *GhKWL1* significantly increased cotton resistance to Verticillium wilt.

## 2. Results

### 2.1. GhKWL1 Is Induced by V. dahliae and the Protein Is Located in the Nucleus

**Figure 1:**
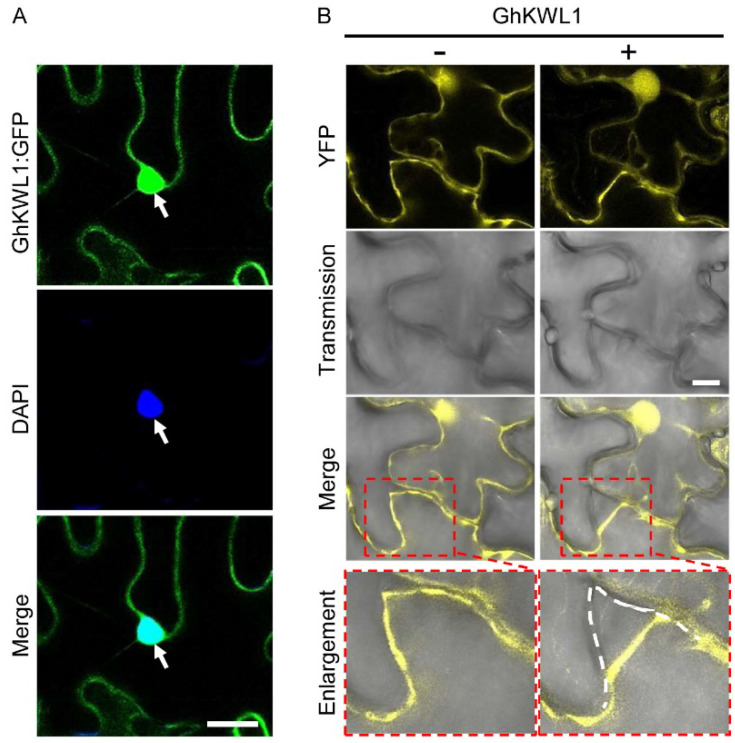
The subcellular localization of GhKWL1. (**A**) Colocalization of the GhKWL1:GFP fusion protein with DAPI-stained nucleus in tobacco (*Nicotiana benthamiana*) pavement cells. *Agrobacterium tumefaciens* infiltration solution containing the *pro35S*:*GhKWL1*:*GFP* vector was infiltrated into tobacco leaves. After 3 d, the infiltrated leaves were stained with DAPI for 20 min, and the fluorescence signals were observed. The white arrows indicate the nucleus. DAPI, a fluorescent dye of DNA. Merge, overlay of green and blue fluorescence images. (**B**) Subcellular localization of GhKWL1:YFP after plasmolysis. *Agrobacterium tumefaciens* infiltration solution containing the *pro35S*:*GhKWL1*:*YFP* vector was infiltrated into tobacco leaves. After 3 d, the infiltrated leaves were imaged (−) and then treated with 30% (*w*/*v*) sucrose solution (+) for plasmolysis. Merge, overlay of fluorescence and transmission images. Regions enclosed in the red boxes are enlarged in the bottom panel. The white dotted line represents the cell wall. Scale bars represent 50 μm (in (**A**)) and 25 μm (in (**B**)).

We used KWL protein sequences of *Z. mays* and *A. chinensis* to query the reference genome of *G. hirsutum* [50]. A total of 35 homologous genes were identified, of which 13 genes originated from the A subgenome and 22 genes from the D subgenome (Supplementary Materials, Figure S1). These *GhKWLs* were expressed globally in the roots, stems, and leaves with various transcription levels (Supplementary Materials, Figure S2A). After a challenge with *V. dahliae* strain V991, four *GhKWL* genes (*GhKWL1*, *GhKWL2*, *GhKWL3*, and *GhKWL4*) were remarkably upregulated (Supplementary Materials, Figure S2B), suggesting that these four genes may be involved in the response of cotton to *V. dahliae* infection. A multiple sequence alignment of ZmKWL1, AcKWL1 [38,42,48], and GhKWL1 to GhKWL4 revealed that the protein sequences were relatively conserved in the central and C-terminal regions but divergent in the N-terminal tails (Supplementary Materials, Figure S3). The prediction of subcellular localization suggested that GhKWL1 might be a nuclear or cytoplasmic protein, GhKWL4 a nuclear or apoplastic protein, and GhKWL2 and 3 might be apoplastic proteins (Supplementary Materials, Table S1). To verify the localization, the four *GhKWL* genes were fused with the green fluorescent protein gene (*GFP*) at the C-terminal, and the *GFP*-tagged genes were transformed into leaves of tobacco (*Nicotiana benthamiana*) using *Agrobacterium*-mediated infiltration. The fusion protein GhKWL1:GFP was mainly localized in the nucleus and at the periphery of pavement cells (Figure 1A), whereas the other three fusion proteins, including GhKWL4:GFP, were localized at the cell periphery, and no nuclear localization was observed (Supplementary Materials, Figure S4). Plasmolysis further indicated that GhKWL1:YFP was not located in the apoplast (Figure 1B).

### 2.2. Downregulation of GhKWL1 Decreases, While Overexpression of GhKWL1 Increases, Resistance to V. dahliae Infection

**Figure 2:**
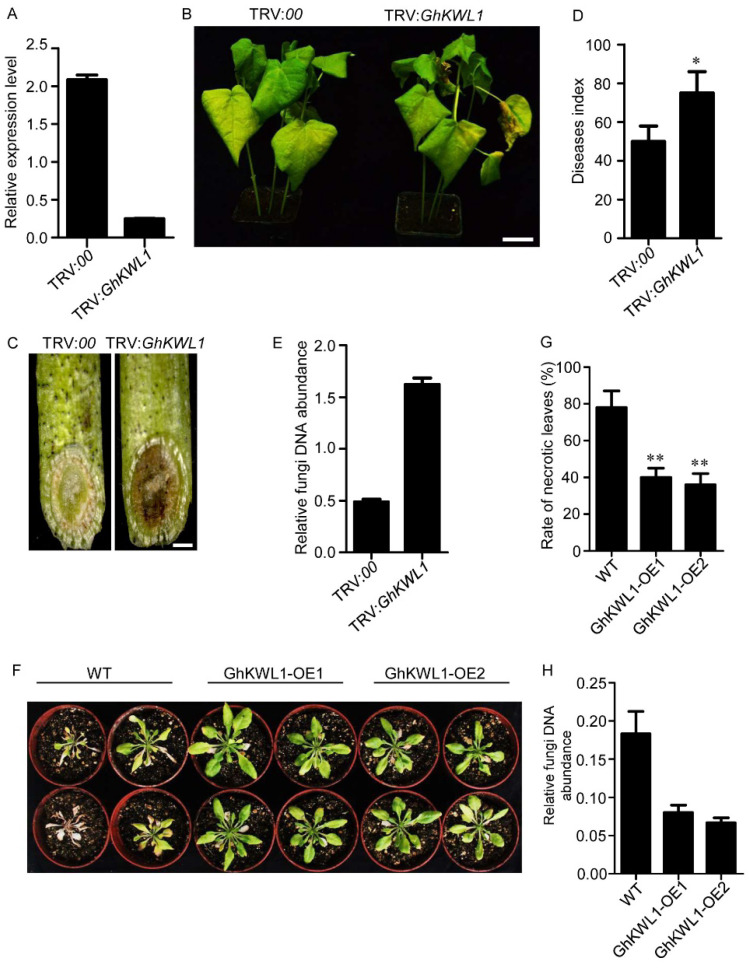
The downregulation of *GhKWL1* enhances the susceptibility of cotton to *V. dahliae* infection, and the overexpression of *GhKWL1* enhances the resistance of Arabidopsis to *V. dahliae* infection. (**A**) The expression level of *GhKWL1* in leaves of *GhKWL1*-silenced (TRV:*GhKWL1*) plants and the parallel control (TRV:*00*). Total RNAs were extracted from leaves of cotton plants after 2 weeks of virus-induced gene silencing of *GhKWL1*. (**B**) Disease symptoms of TRV:*00* and TRV:*GhKWL1* cotton plants infected with *V. dahliae*. Seedlings after 2 weeks of virus-induced gene silencing of *GhKWL1* were inoculated with 10 mL *V. dahliae* conidial suspension (10^7^ conidia/mL) for 20 d. The scale bar represents 3 cm. (**C**) Sections of TRV:*00* and TRV:*GhKWL1* cotton stems at 20 d post-inoculation (dpi). The brown areas are diseased vascular bundles. The scale bar represents 1 mm. (**D**) Disease index of TRV:*00* and TRV:*GhKWL1* cotton plants at 20 dpi. Three independent replicates were performed, with at least 20 plants being measured per VIGS event and replicate. Asterisks indicate statistical significance as determined by Student’s *t*-test (* *p* < 0.05). (**E**) Fungal DNA abundance in leaves of TRV:*00* and TRV:*GhKWL1* cotton plants. The DNA was isolated from upland cotton leaves at 20 dpi. (**F**) Disease symptoms of Arabidopsis infected with *V. dahliae*. Three-week-old Arabidopsis plants were inoculated with *V. dahliae* for 25 d. Three independent replicates were performed, with at least 20 plants being measured per transgenic event and replicate. (**G**) Percentage of necrotic leaves in the Arabidopsis plants at 25 dpi. Asterisks indicate statistical significance as determined by Student’s *t*-test (** *p* < 0.01). (**H**) Fungal DNA abundance in leaves of the Arabidopsis plants. The DNA was isolated from Arabidopsis leaves at 25 dpi. The relative level was determined by quantitative RT-PCR (**A**,**E**,**H**). *GhUBQ7* served as the reference gene in cotton (**A**,**E**); *AtACTIN2* served as the reference gene in Arabidopsis (**H**).

We then employed virus-induced gene silencing (VIGS) to examine the role of GhKWL1 in the resistance of cotton to Verticillium wilt. Two weeks after infiltration, the transcription level of *GhKWL1* was significantly reduced by ~90% compared with that of the control (Figure 2A). The plants were then inoculated with *V. dahliae* strain V991 for 20 d. The knockdown of *GhKWL1* resulted in disease symptoms of increased severity, including wilting, chlorosis, and necrosis, as well as xylem browning than those of the parallel control (Figure 2B,C). Statistically, the disease index of TRV:*GhKWL1* plants was significantly higher than that of the control (Figure 2D). The estimation of the fungal biomass in the leaves by quantitative PCR indicated that more fungi colonized the *GhKWL1*-silenced plants than the control (Figure 2E). Downregulation of *GhKWL1* suggested that the gene is involved in the resistance of plants to Verticillium wilt.

To confirm the role of GhKWL1 in the resistance to Verticillium wilt, we expressed the gene under the control of the *Cauliflower mosaic virus* 35S promoter (35S) in Arabidopsis. Two lines (T_3_ generations) derived from independent transformants that showed a high expression level of *GhKWL1* were selected for further study (Supplementary Materials, Figure S5). Three-week-old plants were inoculated with a conidial suspension of *V. dahliae* strain V991. After 25 d, wilting, chlorosis, and necrosis symptoms were observed in the wild-type plants (Figure 2F). The *35S*:*GhKWL1* Arabidopsis transformants exhibited much less severe symptoms: the plants were larger than the wild type, and few leaves turned yellow (Figure 2F). The percentage of necrotic leaves of *35S*:*GhKWL1* Arabidopsis transformants was significantly lower than that of the wild-type control (Figure 2G). The abundance of fungal DNA in the *GhKWL1*-overexpressing leaves was significantly lower than that of the control (Figure 2H). These results indicate that heterologous overexpression of *GhKWL1* can increase the resistance of Arabidopsis to *V. dahliae* infection.

### 2.3. GhKWL1 Upregulates Transcription of GhERFs

**Figure 3:**
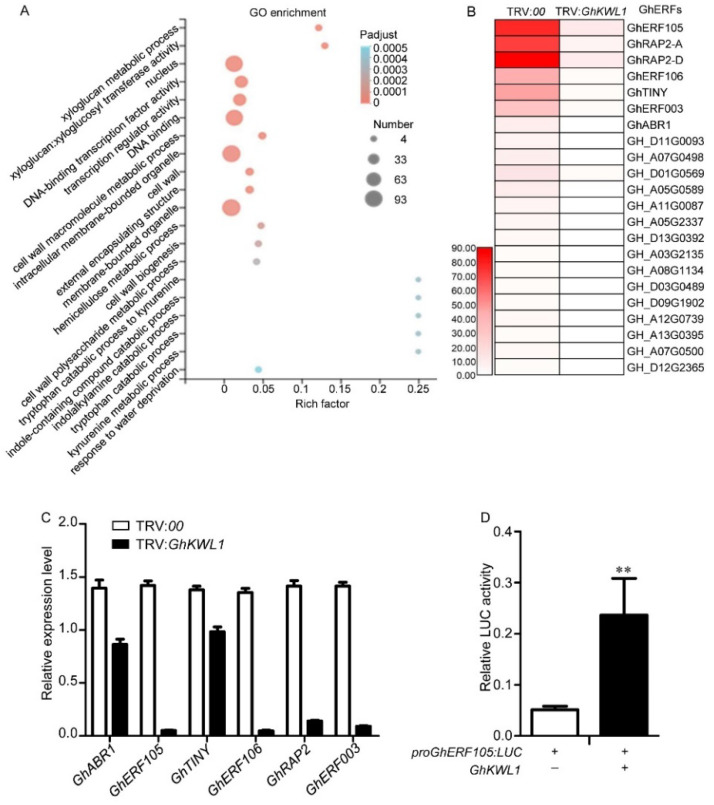
GhKWL1 plays a positive role in upregulating the expression of *GhERF* genes. (**A**) Gene ontology (GO) enrichment of downregulated genes in *GhKWL1*-silenced plants (TRV:*GhKWL1*) vs. the control (TRV:*00*). The *x*-axis represents the Rich Factor. The greater the value, the greater the enrichment degree. The *y*-axis represents various GO terms. The bubble size indicates the number of differential genes in each GO term. The color code indicates the P adjust value. If the *p* adjust value <  0.05, the pathway was defined as a significantly enriched GO term. (**B**) Heat map of the relative expression level of *GhERF* genes (determined by RNA-seq reads) in *GhKWL1*-silenced plants and the control (TRV:*00*). Total RNAs were extracted from leaves of cotton plants after 2 weeks of virus-induced gene silencing of *GhKWL1* and used for RNA-seq analysis. (**C**) Relative expression levels of *GhERF* genes in *GhKWL1*-silenced (TRV:*GhKWL1*) plants and the control (TRV:*00*). Total RNAs were extracted from leaves of cotton plants after 2 weeks of virus-induced gene silencing of *GhKWL1*. The relative level was determined by quantitative RT-PCR. *GhUBQ7* served as the reference gene. GhERF105 represents GH_A03G0725; GhRAP2-A represents GH_A05G1425; GhRAP2-D represents GH_D05G1440; GhERF106 represents GH_D07G0504; GhTINY represents GH_D10G1125; GhERF003 represents GH_D05G0585; GhABR1 represents GH_A02G1617. (**D**) The promoter of *GhERF105* was activated by the expression of *GhKWL1*. The luciferase activity was determined by the LUC/REN ratio. Asterisks indicate statistical significance as determined by Student’s *t*-test (** *p* < 0.01).

An autoactivation activity assay showed that the complete GhKWL1 protein possessed strong autoactivation activity (Supplementary Materials, Figure S6). The nuclear localization of GhKWL1 and the autoactivation activity of GhKWL1 imply that this protein may participate in regulating genes involved in the defense response against *V. dahliae* infection. To understand how GhKWL1 confers resistance to *V. dahliae* infection, we used transcriptomics to investigate genes that are downregulated in *GhKWL1*-silenced plants vs. the control. In total, 561 genes that showed at least six-fold downregulation were detected, including genes involved in the transcriptional factor-related cellular processes, such as DNA binding transcription activity and transcription regulator activity, were detected (Figure 3A). Among these genes, 22 ethylene response factor (*GhERF*) genes were markedly downregulated in *GhKWL1*-silenced plants (Figure 3B). Quantitative RT-PCR (qRT-PCR) analysis further showed that four of these genes (*GhERF105*, *GhRAP2*, *GhERF106*, and *GhERF003*) were largely repressed compared with expression levels in the control plants (Figure 3C). We then selected *GhERF105*, one of the markedly downregulated genes, to test whether its transcription is directly upregulated by GhKWL1. A dual-luciferase assay showed that, under the expression of *GhKWL1*, the transcriptional activity of *GhERF105* was significantly upregulated (Figure 3D).

### 2.4. Suppression of GhERF105 Increases the Susceptibility of Cotton to V. dahliae Infection

**Figure 4:**
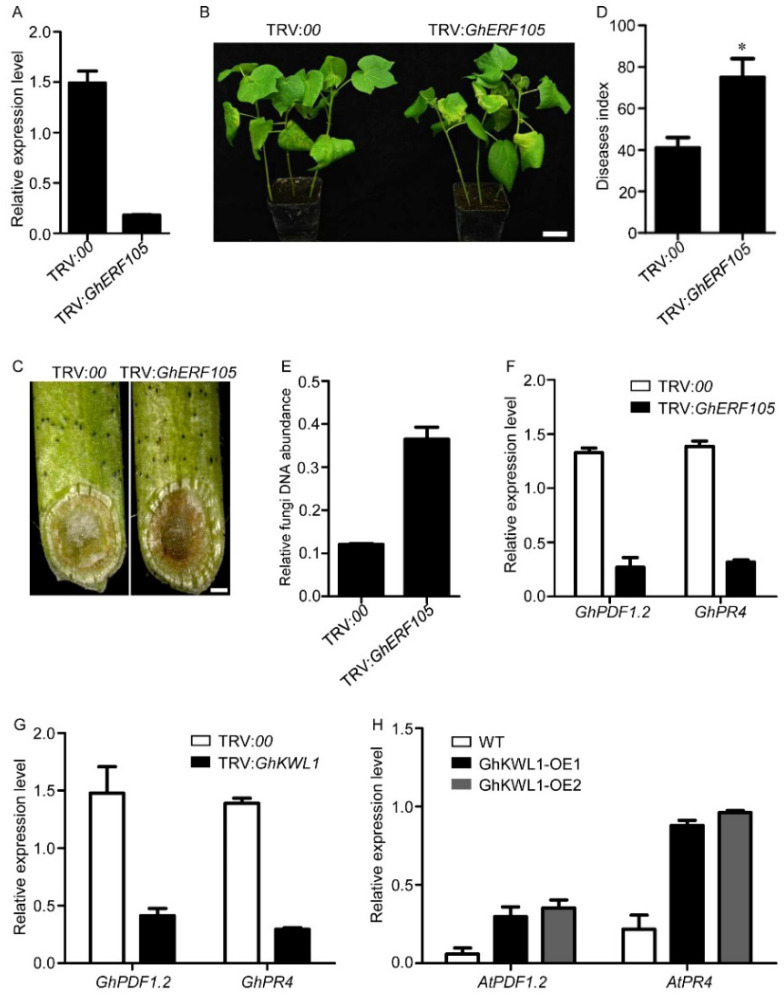
The downregulation of *GhERF105* attenuates cotton resistance to *V. dahliae* infection. (**A**) The expression of *GhERF105* in leaves of cotton plants after 2 weeks of virus-induced gene silencing of *GhERF105*. (**B**) Disease symptoms of cotton plants at 20 d post-inoculation. The roots of cotton seedlings after 2 weeks of virus-induced gene silencing of *GhERF105* were irrigated with 10 mL of *V. dahliae* conidial suspension (10^7^ conidia/mL). The scale bar represents 3 cm. (**C**) Sections of TRV:*00* and TRV:*GhERF105* cotton stems. The brown areas are diseased vascular bundles. The scale bar represents 1 mm. (**D**) The disease index of TRV:*00* and TRV:*GhERF105* plants at 20 dpi. Three independent replicates were performed, with at least 20 plants being measured per VIGS event and replicate. Asterisks indicate statistical significance as determined by Student’s *t*-test (* *p* < 0.05). (**E**) Fungal DNA abundance in leaves of TRV:*00* and TRV:*GhERF105* cotton. The DNA was isolated from cotton leaves at 20 dpi. (**F**) Expression levels of *GhPDF1.2* and *GhPR4* in TRV:*00* and TRV:*GhERF105* cotton plants. Total RNAs were extracted from the cotton leaves after 2 weeks of virus-induced gene silencing of *GhERF105*. (**G**) Expression levels of *GhPDF1.2* and *GhPR4* in TRV:*00* and TRV:*GhKWL1* cotton plants. Total RNAs were extracted from the cotton leaves after 2 weeks of virus-induced gene silencing of *GhKWL1*. (**H**) Expression levels of *AtPDF1.2* and *AtPR4* in wild-type Arabidopsis and *35S*:*GhKWL1* transgenic Arabidopsis lines. Total RNAs were extracted from 3-week-old Arabidopsis leaves. The relative level was determined by quantitative RT-PCR (**A**,**E**–**H**). *GhUBQ7* served as the reference gene in cotton (**A**,**E**,**F**); *AtACTIN2* served as the reference gene in Arabidopsis (**H**).

We then tested whether GhERF105 is involved in plant resistance to *V. dahliae* infection. The expression of *GhERF105* was significantly suppressed by VIGS (Figure 4A). The resultant knockdown cotton plants were infected with *V. dahliae* strain V991. After 20 d, *GhERF105*-silenced plants exhibited worse disease symptoms of chlorosis and xylem browning compared with the control (Figure 4B,C). The disease index and fungal abundance confirmed that the TRV:*GhERF105* plants were more susceptible to infection (Figure 4D,E). It has been reported previously that the expression of *PDF1.2* and *PR4* is positively regulated by ERFs in cotton [15,35]. As expected, transcription of these two genes was decreased in *GhERF105*-silenced or *GhKWL1*-silenced cotton (Figure 4F,G). Conversely, the expression of *AtPDF1.2* and *AtPR4* in *35S*:*GhKWL1* Arabidopsis was noticeably increased (Figure 4H). These results suggested that *PDF1.2* and *PR4* were upregulated by GhKWL1 and GhERF105.

### 2.5. VdISC1 Interacts with GhKWL1 and Inhibits Transcription of GhKWL1

**Figure 5:**
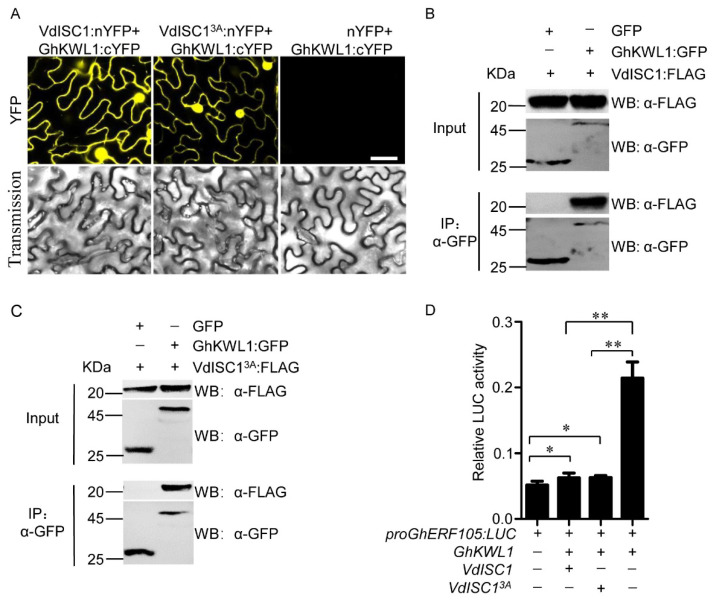
VdISC1 physically interacts with GhKWL1 independent of the catalytic activity of VdISC1. (**A**) VdISC1 or VdISC1^3A^ interaction with GhKWL1 was investigated by BiFC assay. Fusion protein gene *GhKWL1*:*cYFP* was transiently co-expressed with *VdISC1*:*nYFP*, *VdISC1^3A^*:*nYFP,* or *nYFP* in tobacco leaves. The yellow fluorescence signals in tobacco pavement cells were observed at 3 d post-agroinfiltration. The scale bar represents 50 μm. cYFP, the C-terminal half of YFP; nYFP, the N-terminal half of YFP. (**B**) VdISC1 coimmunoprecipitated with GhKWL1. Anti-GFP beads were used to purify the protein complex with GFP-fusion proteins. The total proteins (Input) and anti-GFP immunoprecipitate (IP: α-GFP) were separated by SDS-PAGE and analyzed by immunoblotting with antibodies to FLAG or GFP. The coexpression of *GFP* and *VdISC1*:*FLAG* was used as a negative control. (**C**) VdISC1^3A^ coimmunoprecipitated with GhKWL1. Anti-GFP beads were used to purify the protein complex with GFP-fusion proteins. The total proteins (Input) and anti-GFP immunoprecipitate (IP: α-GFP) were separated by SDS-PAGE and analyzed by immunoblotting with antibodies to FLAG or GFP. The coexpression of *GFP* and *VdISC1^3A^*:*FLAG* was used as a negative control. (**D**) The inducible activity of the *GhERF105* promoter in the presence of GhKWL1, VdISC1, or VdISC1^3A^. The luciferase activity was determined by the LUC/REN ratio. Error bars indicate the standard deviation of three biological replicates. Asterisks indicate statistical significance as determined by Student’s *t*-test (* *p* < 0.05, ** *p* < 0.01).

It was previously reported that ZmKWL1 interacts with UmCmu1, a fungal effector of *Ustilago maydis*, and deactivates the protein to ensure the SA-mediated plant defense response to infection by the pathogen [48]. VdISC1 is an isochorismatase secreted by *V. dahliae* and functions in the same pathway as UmCmu1 to inhibit the conversion of the molecule isochorismate to SA in plants [11]. A bimolecular fluorescence complementation (BiFC) assay showed that both VdISC1 and its loss of catalytic activity mutant VdISC1^3A^ were able to interact with GhKWL1 in the nucleus and endomembrane (Figure 5A). This interaction was further confirmed by a co-immunoprecipitation (Co-IP) assay (Figure 5B,C). In the presence of either VdISC1 or VdISC1^3A^, the activity of the *GhERF105* promoter caused by GhKWL1 was strikingly decreased (Figure 5D). These data suggest that VdISC1 is a negative regulator for the GhKWL1–GhERF105 pathway.

## 3. Discussion

Kiwellins are synthesized in many plant species. However, their functions are poorly understood [48]. To investigate the roles of kiwellins, we identified 35 *KWL* genes in upland cotton. We showed that four *GhKWL* genes were upregulated by *V. dahliae* infection. Among these four GhKWL proteins, only GhKWL1 was shown to be localized in the nucleus. The nuclear localization of GhKWL1 differed from previous reports in which KWLs of kiwifruit and corn were shown to be apoplastic proteins [38,42,48]. Autoactivation activity and dual-luciferase assays revealed that GhKWL1 participates in the activation of *GhERF105*, supporting its nuclear localization.

In dicotyledonous species, SA-mediated defense is associated with resistance to biotrophic pathogens, whereas defense responses mediated by jasmonates (JAs) and ethylene (ET) are implicated in resistance to necrotrophic pathogens. With regard to hemi-biotrophic pathogens, such as *V. dahliae*, the host plants mainly use SA-mediated resistance pathways as defense against the invasion of the pathogen (during the biotrophic phase) and then adopt JA/ET-mediated defenses to limit the pathogen from spreading (during the necrotrophic phase). Among KWL proteins, only ZmKWL1 and ZmKWL1-b were shown to function in disease resistance [48,49]. The resistance mechanism of ZmKWL1 acts via interaction with Cmu1 to deactivate the pathogenic effector [48]. In the present study, we demonstrated that silencing of *GhKWL1* decreased the resistance, whereas overexpression of *GhKWL1* enhanced the resistance of plants to *V. dahliae*. The present data further showed that GhKWL1 could interact with VdISC1, a pathogenic effector produced by *V. dahliae*. Similar to Cmu1, VdISC1 can weaken plant immunity by reducing the production of SA [11]. However, unlike ZmKWL1 and ZmKWL1-b, which are located in the apoplast, GhKWL1 was localized in the nucleus, suggesting it plays a potential role in regulating gene expression. We further revealed that GhKWL1 could upregulate the expression of *ERF* genes. Previous studies have shown that ERFs positively regulate *PDF1.2* and *PR4*, which are associated with resistance to Verticillium wilt, in cotton [15,35]. The decreased expression levels of *PDF1.2* and *PR4* in *GhKWL1*-silenced and *GhERF105*-silenced upland cotton plants, and the enhanced expression levels of the two genes in *GhKWL1*-overexpression Arabidopsis plants, demonstrate that *PDF1.2* and *PR4* are downstream genes of GhKWL1–GhERF105-mediated transcriptional regulation. *PDF1.2* and *PR4* are typical JA-inducible and ET-inducible genes [51,52]. Accordingly, we suggest that VdISC1 has dual pathogenic functions to subvert the immunity of upland cotton: inhibiting SA biosynthesis by decreasing the accumulation of SA precursors [11] during the biotrophic phase of infection and suppressing the expression of JA/ET-inducible resistance genes by binding with GhKWL1, the up-regulator of *ERF* genes, during the necrotrophic phase of infection.

**Figure 6:**
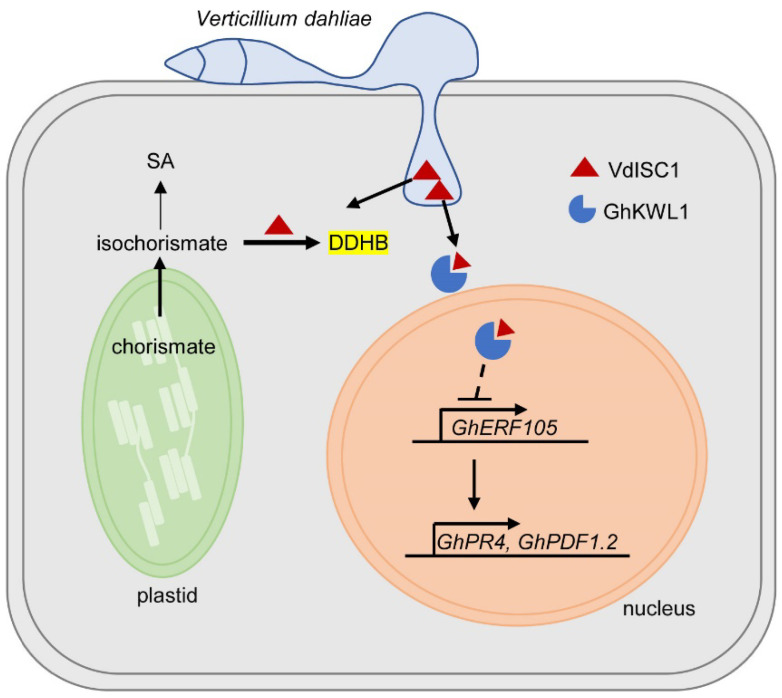
A schematic model of the *Verticillium dahliae* effector VdISC1 disturbing the plant immune system by two distinct scenarios. (i) VdISC1 (red triangle) interacts with GhKWL1 (blue circle missing a wedge) to inhibit GhKWL1-mediated defense response. (ii) Due to its isochorismate mutase activity, VdISC1 converts isochorismate into 2,3-dihydro-2,3-dihydroxybenzoate (DDHB) to inhibit SA accumulation [11].

Taken together, these results indicate that VdISC1 not only converts isochorismate into DDHB to inhibit SA accumulation [11] but also interacts with GhKWL1, a positive regulator in the resistance of cotton against Verticillium wilt, to suppress the function of GhKWL1 in regulating the expression of genes relevant to the defense response (Figure 6). The upregulation of *GhKWL1*, in turn, ensures that the biosynthesis of SA proceeds at considerable levels and protects the GhKWL1–GhERFs pathway from inhibition, thus increasing resistance to Verticillium wilt.

## 4. Materials and Methods

### 4.1. Vector Construction and Plant Materials

The coding sequences (without the stop codon) of *GhKWL1*, *GhKWL2*, *GhKWL3*, and *GhKWL4* were amplified from root cDNA of upland cotton ‘Jimian 14’ with the flanking sites *BamH*I and *Sal*I. *GFP* was amplified with the flanking sites *Sal*I and *EcoR*I; then, the *GFP* fragment was fused at the C-terminal of these *GhKWL* fragments in the binary vector pLGN [53] by *BamH*I and *EcoR*I, respectively. For the *pro35S*:*GhKWL1*:*YFP* construct, the coding sequence (without the stop codon) of *GhKWL1* was amplified with the flanking sites *Kpn*I and *EcoR*I; *YFP* was amplified with the flanking sites *EcoR*I and *BamH*I. The *YFP* fragment was then fused to the 3′ terminal region of *GhKWL1* and inserted into the pLGN vector at the *Kpn*I and *BamH*I sites. For TRV constructs, the 200 bp sequence, which included the 3′-untranslated region of *GhKWL1* and *GhERF105*, were amplified from upland cotton and separately integrated into the TRV2 plasmid using *Kpn*I and *EcoR*I to construct VIGS-related vectors. A partial cDNA sequence of *GhCLA1*, which encodes 1-deoxy-D-xylulose-5-phosphate synthase, was cloned into the TRV2 vector as described [15]. The 1830 bp promoter sequence of *GhERF105* was amplified from upland cotton ‘Jimian 14’ and cloned into the pGreenII 0800-*LUC* vector [54] between the *Hind*III and *BamH*I sites. The coding region of *VdlSC1* was amplified from cDNA of *V. dahliae* strain V991 and inserted into the pLGN vector using *Sal*I and *EcoR*I to construct *pro35S*:*VdlSC1*. For the *pro35S*:*VdlSC1^3A^* construct, the mutated version of *VdlSC1^3A^* (D25A, K90A, and C124A) was synthesized with the flanking *Kpn*I and *EcoR*I restriction sites and inserted into the pLGN vector. For the *pro35S*:*GhKWL1* construct, the coding region of *GhKWL1* was inserted into the pLGN vector using *BamH*I and *EcoR*I. For the BiFC constructs, the coding region of *GhKWL1* (without the stop codon) was fused with *nYFP* in the plasmid pEarleyGate201; the coding region of *VdlSC1* (without the stop codon) and the mutated version of *VdlSC1^3A^* were separately fused with *cYFP* in the plasmid pEarleyGate202 [55]. The coding sequence of *GhKWL1* was cloned into the pLexA vector at the *EcoR*I and *BamH*I sites. The sequence for a FLAG tag was added to the 3′ end of *VdlSC1* (*VdlSC1*:*FLAG*) using PCR amplification and ligated into the pLGN vector at the *BamH*I and *EcoR*I sites. All constructs except pLexA-*GhKWL1* were introduced into *A. tumefaciens* strain GV3101 for further research. Transformation of *A. thaliana* ecotype Columbia-0 was performed using the floral dip method [56]. The sequences of all primers are listed in Supplementary Materials, Table S2. The vectors used are listed in Supplementary Materials, Table S3.

### 4.2. Sequence Retrieval, Phylogenetic Analysis, Subcellular Localization Prediction and Sequence Alignment

The maize and kiwifruit genome sequences were obtained from the Kiwifruit Genome Database (http://kiwifruitgenome.org/, accessed on 19 January 2020) and maizeGDB (https://maizegdb.org/, accessed on 20 January 2020), respectively. The genome sequences of *Oryza sativa* (subsp. *japonica*) were obtained from the Rice Genome Annotation Project (http://rice.plantbiology.msu.edu/, accessed on 13 April 2021), the tobacco genome sequences were obtained from the National Center for Biotechnology Information databases (https://www.ncbi.nlm.nih.gov/, accessed on 13 April 2021). The BLASTP tool was used to identify KWL homologs using ZmKWL and AcKWL sequences with default parameters in the CottonFGD database (https://www.cottonfgd.org/, accessed on 25 January 2020) [50,57]; the cut-off value was 1.0^−50^. The selected cotton KWL proteins were used for further identification of KWLs by searching the cotton database again. All obtained sequences were sorted as unique sequences, and further protein domain searches were performed using InterProScan (http://www.ebi.ac.uk/Tools/pfa/iprscan/, accessed on 26 January 2020). A phylogenetic tree of deduced KWL amino acids was constructed using the neighbor-joining algorithm with default parameters, with 1000 bootstrap replicates as implemented in MEGA6.0 (https://www.megasoftware.net/, accessed on 14 April 2021).

The subcellular localization of GhKWLs was predicted using WoLF PSORT (https://www.genscript.com/tools/wolf-psort/, accessed on 15 April 2021), PSORT (http://psort1.hgc.jp/form.html/, accessed on 15 April 2021), and Localizer (http://localizer.csiro.au//, accessed on 15 April 2021).

The KWL protein sequences were aligned using Megalign™ DNAstar [58] and analyzed with the Gendoc software [59].

### 4.3. Transient Expression in Tobacco Epidermal Cells and BiFC Assay

The overnight culture of *Agrobacterium* was resuspended and diluted with infiltration buffer (10 mM MgCl_2_ and 100 μM acetosyringone) to OD_600_ of 0.01–0.05 and then infiltrated into 8-week-old *N. benthamiana* leaves for transient expression. After growth for 3 d at 25 °C, the infiltrated leaves were harvested for the following analysis.

The BiFC assay was performed as previously described [55]. *Agrobacterium tumefaciens* infiltration solution containing the pEarleyGate201-*pro35S*:*GhKWL1*:*cYFP* and pEarleyGate202-*pro35S*:*VdISC1*:*nYFP* constructs was used to test the interaction between GhKWL1 and VdISC1. *Agrobacterium tumefaciens* infiltration solution containing the pEarleyGate201-*pro35S*:*GhKWL1*:*cYFP* and pEarleyGate202-*pro35S*:*VdISC13A*:*nYFP* constructs was used to test the interaction between GhKWL1 and VdISC1^3A^. *Agrobacterium tumefaciens* infiltration solution containing the pEarleyGate201-*pro35S*:*GhKWL1*:*cYFP* and pEarleyGate202-*pro35S*:*nYFP* constructs were used as a negative control. The interaction was evaluated 3 d after infiltration.

### 4.4. Microscopy Observation

The fluorescence signal was observed using a Leica SP8 confocal microscope equipped with a HyD detector under a 40× oil immersion objective lens. The nucleus was stained with DAPI (4′,6-diamidino-2-phenylindole), a fluorescent dye that strongly binds to DNA. The acquisition parameters were: GFP, excitation at 488 nm, emission from 496 to 530 nm; YFP, excitation at 514 nm, emission from 526 to 570 nm; RFP, excitation at 552 nm, emission from 558 to 606 nm; and DAPI, excitation at 405 nm, emission from 420 to 464 nm.

### 4.5. Quantitative RT-PCR Analysis

The total RNA was extracted using the EASYspin Plant RNA Extraction Kit (Aidlab, Beijing, China). After treatment with DNase I (TaKaRa, Kusatsu, Shiga, Japan), 1 µg RNA was used to synthesize the first-strand cDNA with the PrimeScript™ RT Reagent Kit (TaKaRa, Kusatsu, Shiga, Japan). Quantitative PCR was performed on a CFX96™ Real-Time System (Bio-Rad, Hercules, CA, USA) using the 1× iQ™ SYBR Green Supermix (Bio-Rad, Hercules, CA, USA) in accordance with the manufacturer’s instructions, and the data were analyzed using the native software (Bio-Rad, Hercules, CA, USA). The thermal cycling protocol consisted of a pretreatment (94 °C, 3 min) followed by 40 amplification cycles (94 °C, 30 s; 56 °C, 30 s; and 72 °C, 30 s). The data were analyzed using the CFX Maestro software installed on the CFX96™ Real-Time System (Bio-Rad, Hercules, CA, USA). Each test was confirmed by three individual runs (biological replicates), and data from one of the replicates was used to generate the expression chart. The ubiquitin gene *UBQ7* (GenBank accession no. DQ116441) and actin gene *ACTIN2* (AT3G18780.2) served as the reference genes in cotton and Arabidopsis, respectively. Gene-specific primers used for qRT-PCR are listed in Supplementary Materials, Table S2.

### 4.6. Virus-Induced Gene Silencing in Cotton and RNA-Seq Analysis

*Agrobacterium tumefaciens* infiltration solutions containing the corresponding TRV constructs were prepared according to the previous description [60]. Ten-day-old seedlings of upland cotton were used for infiltration and then grown in a growth chamber under a 16-h-day/8-h-night photoperiod at 25 °C. After 2 weeks, the silenced plants were used for further analysis, including RNA-seq analysis and inoculation with *V. dahliae*. The albino phenotype of *GhCLA1*-silenced cotton plants was used as an indicator of VIGS effectivity in each experiment.

Leaves from more than three *GhKWL1*-silenced plants and the control that was infiltrated with the empty vector were harvested for RNA-seq analysis. The analysis was conducted by Majorbio (http://www.majorbio.com/, accessed on 23 July 2020). The data were analyzed using the Majorbio online analysis system (http://www.majorbio.com/, accessed on 24 July 2020). Genes that showed six-fold downregulation (*GhKWL1*-silenced plants vs. the control) were screened out and enriched by gene ontology (GO) enrichment analysis.

### 4.7. Inoculation Method and Disease Assays

The defoliating *V. dahliae* isolate V991, originally isolated from an infected upland cotton plant, was used in this study [61]. For the production of a conidial suspension, mycelia were grown on potato dextrose agar medium, then collected and cultured in liquid Czapek’s medium. Disease assays with *V. dahliae* were performed as previously described [62]. Upland cotton and Arabidopsis plants in pots were inoculated by drenching the soil with 10 mL conidial suspension (1 × 10^7^ conidia/mL). Verticillium wilt symptoms were recorded for three inoculated plants, with each gene target harvested at 20 d post-inoculation (dpi) in cotton and 25 d post-inoculation (dpi) in Arabidopsis. The diseased cotton seedlings were classified into five grades (0, 1, 2, 3, and 4) based on the severity of the disease after infection. The disease index was calculated using the formula: Disease index (%) = (∑(disease grades × number of infected plants)/(total number of plants × 4)) × 100. The quantification of the *V. dahliae* biomass in the plant was conducted in accordance with previous reports [63,64]. Stems of three inoculated plants were harvested at 20 dpi for cotton, and leaves of three inoculated plants were harvested at 25 dpi for Arabidopsis. The samples were ground to powder, and genomic DNA was isolated. Analysis by qRT-PCR was conducted using the fungus-specific primer ITS-F in combination with the *V. dahliae*-specific reverse primer ST-VE1-R. Cotton *GhUBQ7* (GenBank accession no. DQ116441) and Arabidopsis *AtACTIN2* (AT3G18780.2) were used as endogenous plant controls. For the expression analysis of *GhKWL* genes in upland cotton after inoculation with *V. dahliae* V991, 3-week-old seedlings of *G. hirsutum* ‘Jimian 14’ were inoculated by drenching the soil with 10 mL conidial suspension (1 × 10^7^ conidia/mL). The inoculated leaf samples were collected at four time points (12, 24, 36, and 48 h) post-inoculation, with three seedlings collected per sample. Control plants were treated with sterile distilled water (0 h).

### 4.8. Dual-Luciferase Reporter Assay

The dual-luciferase reporter assay was performed as previously described [54]. The promoter sequence of *GhERF105* was cloned into the pGreenII 0800-*LUC* vector, and the coding region of *GhKWL1*, *VdISC1*, and *VdISC1^3A^* was cloned into the pLGN vector. Dual-luciferase reporter activities were measured using the Dual-Glo^®^ Luciferase Assay System (Promega, Madison, Wisconsin, USA).

### 4.9. Co-Immunoprecipitation Assay

For the Co-IP assay, *A. tumefaciens* infiltration solution containing the pLGN-*pro35S*:*GhKWL1*:*GFP* and pLGN-*pro35S*:*VdISC1*:*FLAG* constructs was used to test the interaction between GhKWL1 and VdISC1; the infiltration solution containing the pLGN-*pro35S*:*GFP* and pLGN-*pro35S*:*VdISC1*:*FLAG* constructs was used as the negative control. *Agrobacterium tumefaciens* infiltration solution containing the pLGN-*pro35S*:*GhKWL1*:*GFP* and pLGN-*pro35S*:*VdISC1^3A^*:*FLAG* constructs was used to test the interaction between GhKWL1 and VdISC1^3A^; the infiltration solution containing the pLGN-*pro35S*:*GFP* and pLGN-*pro35S*:*VdISC1^3A^*:*FLAG* constructs was used as the negative control. After 3 d, approximately 1 g infiltrated leaves of *N. benthamiana* were ground in liquid nitrogen and extracted in 3 mL extraction buffer (94.7 mL of 0.2 M Na_2_HPO_4_, 5.3 mL of 0.2 M NaH_2_PO_4_, 1 g polyvinylpyrrolidone, and 1 mL β-mercaptoethanol) supplemented with 0.1% protease inhibitor cocktail (Thermo Scientific, Rockford, IL, USA, Catalog number 87786). After centrifugation at 20,000 rpm for 10 min, the supernatant was the crude extract of the total protein. For immunoprecipitation, 25 μL Anti-GFP mAb-Magnetic beads (MBL, Beijing, China, Catalog number D153-10) was added to 3 mL of the total protein and incubated at 4 °C for 1 h in accordance with the manufacturer’s instructions. Proteins immunoprecipitated with the agarose beads were then separated in 10% SDS-PAGE gel and transferred to a PVDF membrane for western blot analysis. The GFP-fusion protein was detected using a mouse monoclonal antibody against GFP (Thermo Scientific, Rockford, IL, USA, Catalog number MA5-15256) at the dilution 1:1000, and the FLAG-fusion proteins were detected using a mouse monoclonal antibody against FLAG (Thermo Scientific, Rockford, IL, USA, Catalog number MA1-91878) at the dilution 1:1000. A secondary antibody (Thermo Scientific, Rockford, IL, USA, Catalog number 32430), derived from goat and conjugated to horseradish peroxidase, was used for signal detection.

### 4.10. Yeast Autoactivation Assay

The pLexA-GhKWL1 construct was transformed into yeast (*Saccharomyces cerevisiae*) strain EGY48. Transformed cells were grown on a synthetically defined (SD) medium lacking His and Ura for 4 d. Subsequently, positive transformants were mated overnight and spotted onto QDO (quadruple dropouts SD medium lacking Leu, Trp, Ura and His) plates. 5-Bromo-4-chloro-3-indolyl-α-D-galactopyranoside (X-α-gal) was used as a substrate for colorimetric detection of α-galactosidase activity. Plates were incubated at 30 °C for 6–12 d. Transformed yeast cells harboring the pLexA or pLexA-53 and pB42AD-T constructs were used as a negative control and positive control, respectively.

## Data Availability

The data presented in this study are available on request from the corresponding author. The data are not publicly available due to privacy.

## Supplementary Materials

The following are available online at https://www.mdpi.com/article/10.3390/ijms22147328/s1.
