# Supplementary material for: GhKWL1 Upregulates GhERF105 but Its Function Is Impaired by Binding with VdISC1, a Pathogenic Effector of Verticillium dahliae
Source: Int J Mol Sci. 2021 Jul 8;22(14):7328. doi: 10.3390/ijms22147328 (PMC8306359; doi:10.3390/ijms22147328)
Supplement: Supplementary file 1 [file ijms-22-07328-s001.zip › ijms-1264159-supplementary.pdf]

GhKWL1 upregulates *GhERF105* but its function is impaired by binding with VdISC1, a pathogenic effector of *Verticillium dahliae*

Yang Chen [1], Mi Zhang [1], Lei Wang [1], Xiaohan Yu [1], Xianbi Li [1], Dan Jin [1], Jianyan Zeng [1], Hui Ren [1], Fanlong Wang [1], Shuiqing Song [1], Xingying Yan [1], Juan Zhao [1] and Yan Pei [1] *

1 Biotechnology Research Center, Southwest University, No. 2 Tiansheng Road, Beibei, Chongqing, 400716, P. R. China.

*Corresponding author: peiyan3@swu.edu.cn

Biotechnology Research Center, Southwest University, No. 2 Tiansheng Road, Beibei, Chongqing, 400716, P. R. China.

Tel: +86-23-68251883

Fax: +86-23-68250515

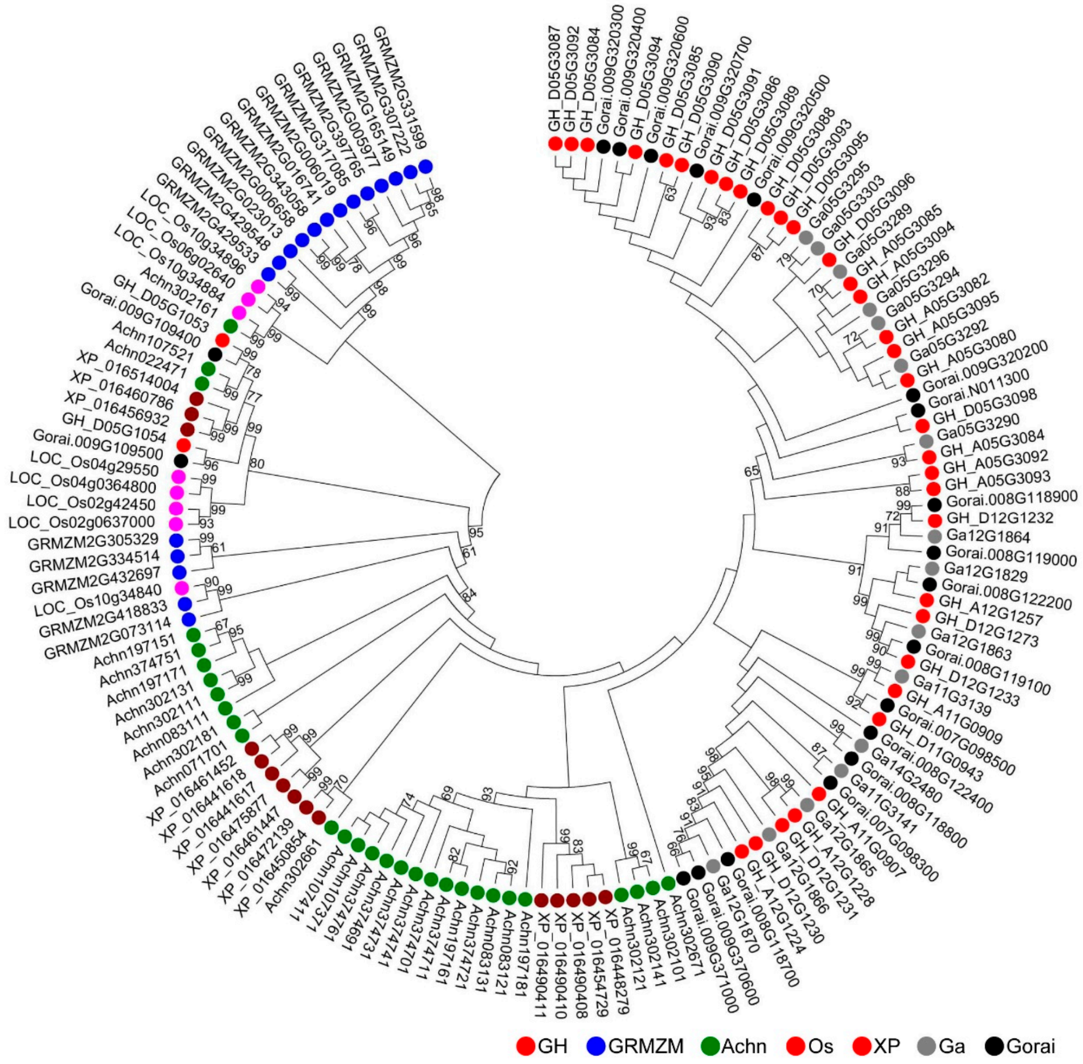

Figure S1. Phylogenetic analysis of KWL proteins in *G. hirsutum*, *G. raimondii, G. arboretum, Z. mays*, *O. sativa* (subsp. *japonica*), *A. chinensis* and *N. tabacum*. The phylogenetic tree was constructed by neighbor-joining method in MEGA6.0. GH, *G. hirsutum*; GRMZM, *Z. mays*; Achn, *A. chinensis*; Os, *O. sativa* (ssp. *japonica*); XP, *N. tabacum*; Ga, *G. arboretum*; Gorai, *G. raimondii*.

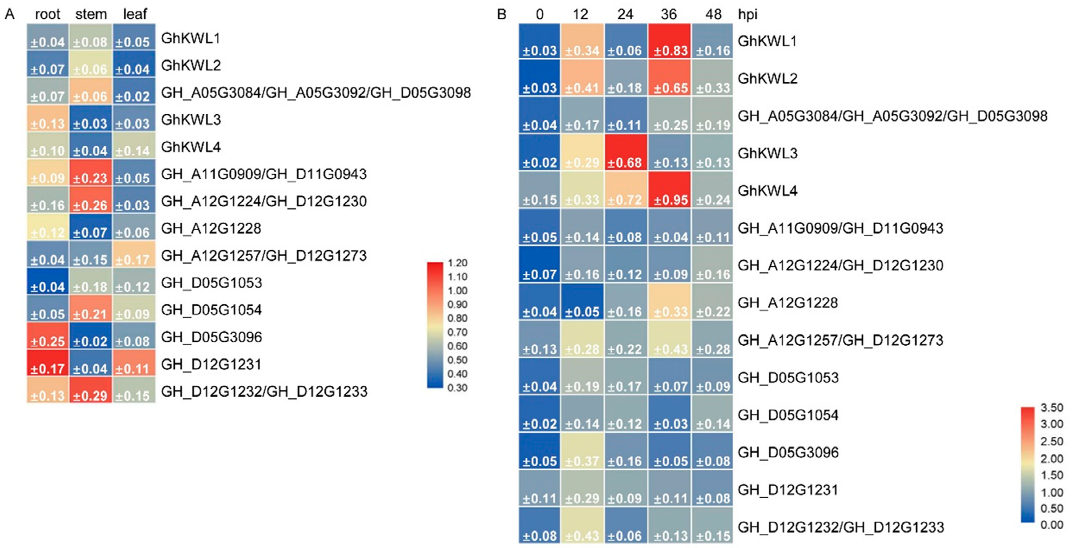

Figure S2. (**A**) Expression profile of *GhKWL* genes in roots, stems and leaves of upland cotton. Total RNAs were extracted from the roots, stems and leaves of upland cotton with two true leaves. *GhUBQ7* served as the reference gene. (**B**) Expression patterns of *GhKWL* genes under *V. dahliae* infection. Total RNAs were extracted from upland cotton leaves at indicated time points (12, 24, 36 and 48 h) after inoculation with *V. dahliae*. Inoculation with sterile distilled water was used as control (0 h). hpi, hours post inoculation. *GhUBQ7* served as the reference gene. The value of number shown in the heatmap represents standard deviation. GhKWL1 represents GH_D05G3086, GH_D05G3089 and GH_D05G3091; GhKWL2 represents GH_D05G3084, GH_D05G3085, GH_D05G3087, GH_D05G3090 and GH_D05G3092; GhKWL3 represents GH_A05G3094; GhKWL4 represents GH_A11G0907.

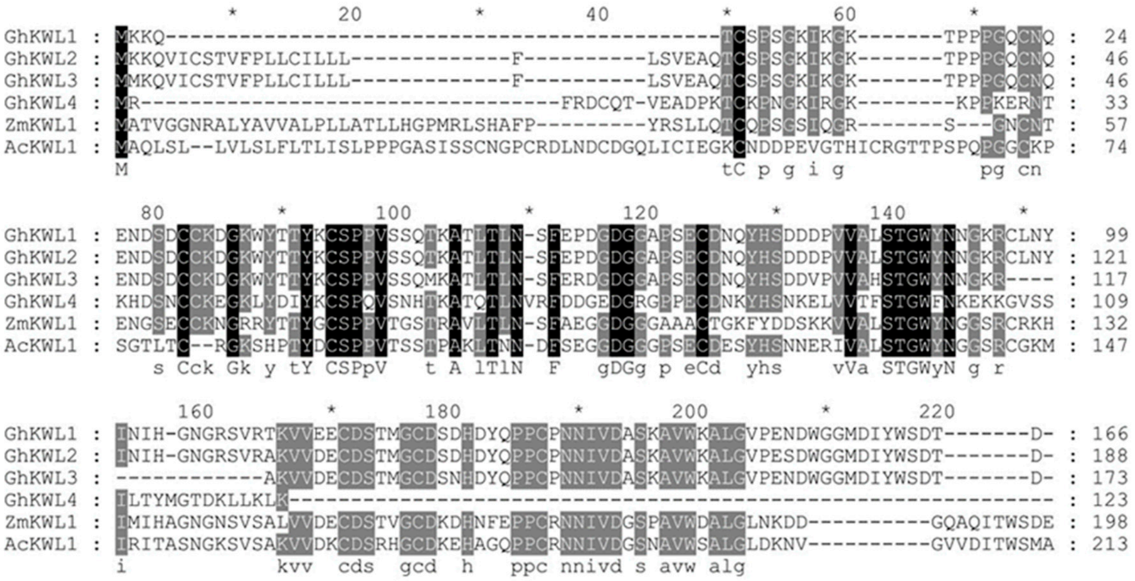

Figure S3. Protein alignment of KWL proteins from upland cotton, maize and kiwifruit. KWL protein sequences were aligned using Megalign^{TM} DNAstar software, and analysed by Gendoc software. GhKWL1, GH_D05G3086; GhKWL2, GH_D05G3092; GhKWL3, GH_A05G3094; GhKWL4, GH_A11G0907; ZmKWL1, GRMZM2G073114; AcKWL1, Achn022471.

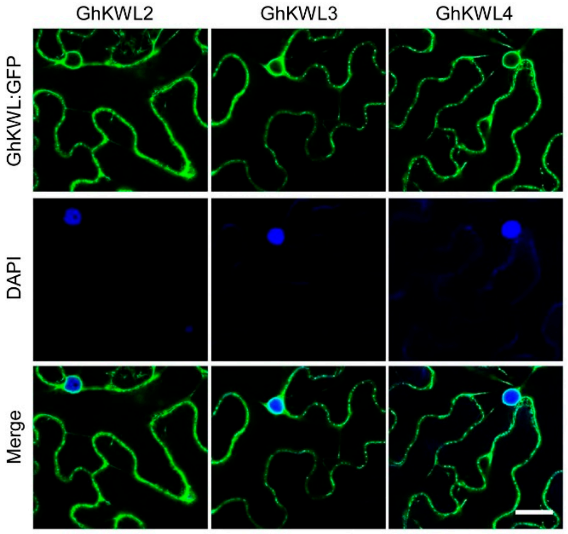

Figure S4. Subcellular localization of GhKWL2:GFP, GhKWL3:GFP, and GhKWL4:GFP fusion proteins. Colocalization of the fusion proteins with DAPI stained nucleus in tobacco (*N. benthamiana*) pavement cells. Scale bars, 50 µm.

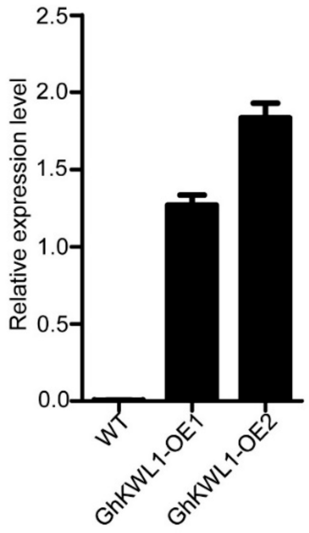

Figure S5. Expression level of *GhKWL1* in transgenic Arabidopsis lines (GhKWL1-OE1 and GhKWL1-OE2). *AtACTIN2* served as the reference gene.

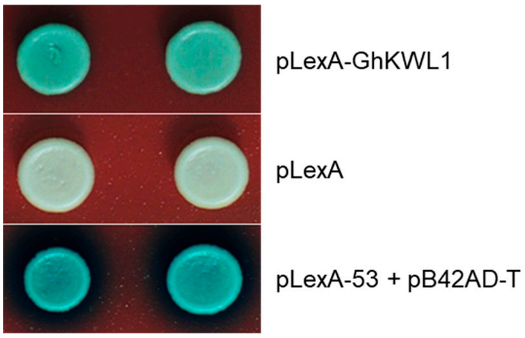

Figure S6. Transcription autoactivation of GhKWL1. The whole coding region of *GhKWL1* was used for transcription autoactivation assay. Transformed yeast cells harboring pLexA or pLexA-53 + pB42AD-T constructs were used as negative and positive control, respectively.

Table S1. Prediction of subcellular localization of GhKWLs.

Tools	Predicted location			
	GhKWL1	GhKWL2	GhKWL3	GhKWL4
PSORT	cytoplasm	Apoplast	Apoplast	Apoplast
Localizer	-	-	-	Nucleus
WoLF PSORT	Nucleus	Extracellular	Extracellular	Nucleus

Dashes represent no subcellular localization was predicted.

Table S2. Primers used in this study.

Primer code	Sequence, 5’-3’	Description
GhKWL1-F(*BamHI*)	ggatccATGAAGAAGCAAACGTGCAG	GhKWL1 without stop codon
GhKWL1-R(*SalI*)	gtcgacATCAGTATCAGACCAGTAGAT	GhKWL1 without stop codon
GhKWL2-F(*BamHI*)	ggatccATGAAGAAGCAAGTAATTTG	GhKWL2 without stop codon
GhKWL2-R(*SalI*)	gtcgacATCAGTATCAGACCAGTAGA	GhKWL2 without stop codon
GhKWL3-F(*BamHI*)	ggatccATGATGAAGCAAGTAATTTG	GhKWL3 without stop codon
GhKWL3-R(*SalI*)	gtcgacATCAGTATCAGACCAGTAGAT	GhKWL3 without stop codon
GhKWL4-F(*BamHI*)	ggatccATGAGATTTCGAGACTGTCA	GhKWL4 without stop codon
GhKWL4-R(*SalI*)	gtcgacCTTTAGCTTTAACAGTTTGTC	GhKWL4 without stop codon
GFP-PLGN- F(*SalI*)	gtcgacATGGTGAGCAAGGGCGAGGAG	GFP
GFP-PLGN- R(*EcoRI*)	gaattcTTACTTGTACAGCTCGTCCAT	GFP
GhKWL1-VIGS-F(*KpnI*)	ggtaccGCCCTAACAACATTGTTGAT	GhKWL1 VIGS fragment
GhKWL1-VIGS-R(*EcoRI*)	gaattcATACATAATATATACATAAA	GhKWL1 VIGS fragment
GhERF105-VIGS-F(*KpnI*)	ggtaccTTTAACGCCGTCAAATTGGA	GhERF105 VIGS fragment
GhERF105-VIGS-R(*EcoRI*)	gaattcGGCAATGTACACTAATGATAC	GhERF105 VIGS fragment
GhERF105-pro-F(*HindII*)	aagcttTAAATCCATCTCACTAGGTTG	GhERF105 promoter
GhERF105-pro-R(*BamHI*)	ggatccAAAAAGAGTTGATTTGAGTGG	GhERF105 promoter
VdISC1-PLGN-F(*SalI*)	gtcgacATGTCCTCATTCCGCTCCAT	VdISC1
VdISC1-PLGN-R(*EcoRI*)	gaattcCTACCGTGAGCCAAAAACAGT	VdISC1
VdISC1-3A-PLGN- F(*KpnI*)	ggtaccATGTCCTCATTCCGCTCCAT	$VdISC1^{3A}$
VdISC1-3A-PLGN- R(*EcoRI*)	gaattcCTACCGTGAGCCAAAAACAGT	$VdISC1^{3A}$
GhKWL1-PLGN-F(*BamHI*)	ggatccATGAAGAAGCAAACGTGCAG	GhKWL1
GhKWL1-PLGN-R(*EcoRI*)	gaattcTTAATCAGTATCAGACCAGTA	GhKWL1
GhKWL1-cYFP-F(*AsiSI*)	gcgatcgcATGAAGAAGCAAACGTGCAG	GhKWL1 without stop codon
GhKWL1-cYFP-R(*AsiSI*)	gcgatcgccATCAGTATCAGACCAGTAGAT	GhKWL1 without stop codon
GhKWL1-pLexA-F(*EcoRI*)	gaattcATGAAGAAGCAAGTAATTTG	GhKWL1
GhKWL1-pLexA-R(*BamHI*)	ggatccccATCAGTATCAGACCAGTAGA	GhKWL1
VdISC1-cYFP-F(*AsiSI*)	gcgatcgcATGTCCTCATTCCGCTCCAT	VdISC1 and $VdISC1^{3A}$ without stop codon
VdISC1- nYFP-R(*BamHI*)	ggatccCGTGAGCCAAAAACAGTCGCGA	VdISC1 and $VdISC1^{3A}$ without stop codon
GhKWL1-YFP-F(*KpnI*)	ggtaccATGAAGAAGCAAACGTGCAG	GhKWL1 without stop codon
GhKWL1-YFP-R(*EcoRI*)	gaattcATCAGTATCAGACCAGTAGAT	GhKWL1 without stop codon
YFP-pLGN-F(*EcoRI*)	gaattcATGGGCAAGGGCGAGGAGCT	YFP
YFP-pLGN-R(*BamHI*)	ggatccTCACTTGTACAGCTCGTCCA	YFP
VdISC1-FLAG-F(*KpnI*)	ggtaccATGTCCTCATTCCGCTCCAT	VdISC1 and $VdISC1^{3A}$
VdISC1-FLAG-R(*EcoRI*)	GAATTCctatttgtcgtcatcgtctttgtagtcGTTGATATCCTTGCTCGAGA	VdISC1 and $VdISC1^{3A}$
GhCLA1-TRV-F(*KpnI*)	ggtaccGGGAATTCTTCGGTGACCCTTATAACTCGGACCCGGAG	GhCLA1
GhCLA1-TRV-R(*EcoRI*)	gaattcGGGGATCCATCAAACCGTGCTCTTTCCTCCACAATCTT	GhCLA1
GhKWL1-RT-F	AGATAAAAATGAAGAAGCAAACGT	GhKWL1 qRT PCR in cotton

GhKWL1-RT-R	TCGTGTACCACTTGCCATCCTTG	GhKWL1 qRT PCR in cotton
GhKWL2-RT-F	TTTCTTAGTGTTGAAGCACAG	GhKWL2 qRT PCR
GhKWL2-RT-R	TGTACCACTTGCCATCCTTGCA	GhKWL2 qRT PCR
GhKWL3-RT-F	TTAACAGCTTTGAGCCAGAT	GhKWL3 qRT PCR
GhKWL3-RT-R	CGCACAAGAAATCTTTACTCT	GhKWL3 qRT PCR
GhKWL4-RT-F	AACATGACTCCAATTGCTGC	GhKWL4 qRT PCR
GhKWL4-RT-R	TATCACATTCAGGTGGTCCG	GhKWL4 qRT PCR
GhERF105-RT-F	GAGGAGAAGGGAAGTGTGACGC	GhERF105 qRT PCR
GhERF105-RT-R	ACATAACCGCAAGCCCGGAATA	GhERF105 qRT PCR
GH_A05G3084-RT-F	CCTTGCCCTAACAACATTGTTGA	GH_A05G3084 RT PCR
GH_A05G3084-RT-R	CAGAACAATAGATATCCATCC	GH_A05G3084 RT PCR
GH_A11G0909-RT-F	CATACACGTGTTCACCGCCG	GH_A11G0909 qRT PCR
GH_A11G0909-RT-R	TCACACTCTTTCCATTGCCA	GH_A11G0909 qRT PCR
GH_A12G1224-RT-F	CTCTCAACGGATTCGGTCCGAAG	GH_A12G1224 qRT PCR
GH_A12G1224-RT-R	TCACATTCATCGACTACCTTAGC	GH_A12G1224 qRT PCR
GH_A12G1228-RT-F	TCTCAATGGATTCGATTCTGGT	GH_A12G1228 qRT PCR
GH_A12G1228-RT-R	TGTTAATATACTTCATACACCA	GH_A12G1228 qRT PCR
GH_A12G1257-RT-F	GAGATGGAGGCGGACCAT	GH_A12G1257 qRT PCR
GH_A12G1257-RT-R	GAAAGTGCCACTACAGGTTC	GH_A12G1257 qRT PCR
GH_D05G1053-RT-F	TGCTCACCTCCAGTTACATC	GH_D05G1053 qRT PCR
GH_D05G1053-RT-R	CATTCGTCAACCACCTTAGC	GH_D05G1053 qRT PCR
GH_D05G1054-RT-F	GTGCAATGATGATCCCGATG	GH_D05G1054 qRT PCR
GH_D05G1054-RT-R	AGTGCCACTATACGCTCAAA	GH_D05G1054 qRT PCR
GH_D05G3096-RT-F	TTGTTCGACTGTCTTCCCTC	GH_D05G3096 qRT PCR
GH_D05G3096-RT-R	TTATACCACCCACCATCTCC	GH_D05G3096 qRT PCR
GH_D12G1231-RT-F	CCTCCAGAAGGCAAGTTTTA	GH_D12G1231 qRT PCR
GH_D12G1231-RT-R	CGAATCGAATCCATTGAGAG	GH_D12G1231 qRT PCR
GH_D12G1232-RT-F	TGGAAAAAGCGTTAAGGCTAAG	GH_D12G1232 qRT PCR
GH_D12G1232-RT-R	TTTCTCCCCAATCGTCTTTAG	GH_D12G1232 qRT PCR
GhKWL1OE-RT-F	GAGGTGTTTGAATTATATCAACA	GhKWL1 qRT PCR in Arabidopsis
GhKWL1OE-RT-R	TCCAAGAGCCTTCCAAACTGC	GhKWL1 qRT PCR in Arabidopsis
ITS-F	AAAGTTTTAATGGTTCGCTAAGA	*Verticillium* ribosomal internal transcribed spacer region (ITS) qRT PCR
ST-VE1-R	CTTGGTCATTTAGAGGAAGTAA	*Verticillium* ribosomal internal transcribed spacer region (ITS) qRT PCR
GhUBQ7-RT-F	GAAGGCATTCCACCTGACCAAC	GhUBQ7 qRT PCR
GhUBQ7-RT-R	CAAAACTCCAAAATCATACCCAAAG	GhUBQ7 qRT PCR
AtACTIN2-RT-F	GAACTATGAATTACCCGATG	AtACTIN2 qRT PCR
AtACTIN2-RT-R	TGGAATGTGCTGAGGGAAGC	AtACTIN2 qRT PCR
GhRAP2-RT-F	ACCCTAAGAAGAAGCTGAAAC	GhRAP2 qRT PCR
GhRAP2-RT-R	CCAAAGCTCCAAAGATCCGTTA	GhRAP2 qRT PCR
GhERF106-RT-F	GGCTTATGATTGTGCTGCGTTTA	GhERF106 qRT PCR

GhERF106-RT-R	GTCACTATAACACGTTTTCTAG	GhERF106 qRT PCR
GhERF003-RT-F	GACCAAACAACAATCAGTGCAGC	GhERF103 qRT PCR
GhERF003-RT-R	ACATTGCATATTTCAATGGAG	GhERF103 qRT PCR
GhABR1-RT-F	GTCCCAAGAGAGCAACAACTACA	GhABR1 qRT PCR
GhABR1-RT-R	GTTGGAATCTGATGACCAAGA	GhABR1 qRT PCR
GhTINY-RT-F	CAGGGTAAAAGATGAGGAGGGTC	GhTINY qRT PCR
GhTINY-RT-R	ACTCAAAGCAGGAAGTGAAGA	GhTINY qRT PCR
GhPR4-RT-F	GAGGAGTAAGTATGGTTGGACT	GhPR4 qRT PCR XM_016813433
GhPR4-RT-R	AATCATATCTAATAGTAAGGT	GhPR4 qRT PCR XM_016813433
GhPDF1.2-RT-F	ATCACCCTTATCTTCGCTGCTCTT	GhPDF1.2 qRT PCR XM_016849094
GhPDF1.2-RT-R	TTGACAAACAATATAACATT	GhPDF1.2 qRT PCR XM_016849094
AtPDF1.2-RT-F	ATCACCCTTATCTTCGCTGCTCTT	AtPDF1.2 qRT PCR AT5G44420
AtPDF1.2-RT-R	CATGTTTGGCTCCTTCAAGGTTAA	AtPDF1.2 qRT PCR AT5G44420
AtPR4-RT-F	CCGACCAACAACTGTCAGAGCA	AtPR4 qRT PCR AT3G04720
AtPR4-RT-R	GAGCAATAAGCACTCACGGCTCT	AtPR4 qRT PCR AT3G04720

Table S3. Usages of the vectors we constructed in this study.

Vectors	Description
pLGN-*pro35S:GhKWL1*	Transformation of *A. thaliana* and transient expression of tobacco (Dual-luciferase reporter assay)
pLGN-*pro35S:GhKWL1:GFP*	Transient expression of tobacco (subcellular localization assay and Co-IP assay)
pLGN-*pro35S:GhKWL2:GFP*	Transient expression of tobacco (subcellular localization assay)
pLGN-*pro35S:GhKWL3:GFP*	Transient expression of tobacco (subcellular localization assay)
pLGN-*pro35S:GhKWL4:GFP*	Transient expression of tobacco (subcellular localization assay)
pLGN-*pro35S:VdISC1*	Transient expression of tobacco (Dual-luciferase reporter assay)
pLGN-*pro35S:VdISC1*^{3A}	Transient expression of tobacco (Dual-luciferase reporter assay)
pLGN-*pro35S:GhKWL1:YFP*	Transient expression of tobacco (plasmolysis assay)
pGreenII 0800-*proGhERF105:LUC*	Transient expression of tobacco (Dual-luciferase reporter ass)ay
pLGN-*pro35S:VdISC1:FLAG*	Transient expression of tobacco (Co-IP assay)
pLGN-*pro35S:VdISC1*^{3A}*:FLAG*	Transient expression of tobacco (Co-IP assay)
pLGN-*pro35S:GFP*	Transient expression of tobacco (Co-IP assay)
pEarleyGate202-*pro35S:VdISC1:nYFP*	Transient expression of tobacco (BiFC assay)
pEarleyGate202-*pro35S:VdISC1*^{3A}*:nYFP*	Transient expression of tobacco (BiFC assay)
pEarleyGate201-*pro35S:GhKWL1:cYFP*	Transient expression of tobacco (BiFC assay)
TRV1	Transient expression of cotton (VIGS assay)
TRV:*00*	Transient expression of cotton (VIGS assay)
TRV:*GhCLA1*	Transient expression of cotton (VIGS assay)
TRV:*GhKWL1*	Transient expression of cotton (VIGS assay)
TRV:*GhERF105*	Transient expression of cotton (VIGS assay)
pLexA-*GhKWL1*	Transformation of yeast (yeast autoactivation assay)
pLexA-53	Transformation of yeast (yeast autoactivation assay)
pB42AD-T	Transformation of yeast (yeast autoactivation assay)
